# An intrinsically disordered region of Drosha selectively promotes miRNA biogenesis, independent of tissue-specific Microprocessor condensates

**DOI:** 10.1101/2025.04.10.648254

**Published:** 2025-04-10

**Authors:** Bing Yang, Brian Galletta, Nasser Rusan, Katherine McJunkin

## Abstract

Precise control of miRNA biogenesis is of extreme importance, since mis-regulation of miRNAs underlies or exacerbates many disease states. The Microprocessor complex, composed of DROSHA and DGCR8, carries out the first cleavage step in canonical miRNA biogenesis. Despite recent advances in understanding the molecular mechanism of Microprocessor, the N-terminal region of DROSHA is less characterized due its high intrinsic disorder. Here we demonstrate that Microprocessor forms condensates with properties consistent with liquid-liquid phase separation (LLPS) in select tissues in *C. elegans* . While DRSH-1/Drosha recruitment to granules is only partially dependent on its intrinsically disordered regions (IDRs), one of these N-terminal IDRs is crucial for biogenesis of a subset of miRNAs and normal development. A cis region of an IDR-dependent miRNA confers IDR-dependence to another miRNA, suggesting that the IDR recognizes sequences or structures in the miRNA primary transcript. Future studies will further elucidate the specificity of this interaction and the putative role of Microprocessor condensates.

## Full Text Availability

The license terms selected by the author(s) for this preprint version do not permit archiving in PMC. The full text is available from the preprint server.

